# Supplementary material for: Effectiveness of sofosbuvir based direct-acting antiviral regimens for chronic hepatitis C virus genotype 6 patients: Real-world experience in Vietnam
Source: PLoS One. 2020 May 20;15(5):e0233446. doi: 10.1371/journal.pone.0233446 (PMC7239434; doi:10.1371/journal.pone.0233446)
Supplement: S1 Table — A; non cirrhotic chronic HCV, B; Chronic HCV with compensated cirrhosis (Child Pugh A), C: Chronic HCV decompensated cirrhosis (including moderate and severe liver failure, Child Pugh B or C), D: Doses of treatment. (DOCX) [file pone.0233446.s001.docx]

S1 Table : Treatment regimens and dose following Decision No. 5012/QĐ-BYT by MoH, Vietnam for non cirrhotic chronic HCV, Chronic HCV with compensated cirrhosis (Child Pugh A), and Chronic HCV decompensated cirrhosis (including moderate and severe liver failure, Child Pugh B or C) patients.

a. Non-cirrhotic chronic HCV patients

| **Genotype** | **SOF/LDV** | **SOF/DCV** | **SOF + RBV** | **GZR/EBR** | **SOF/VEL** | **SMV + SOF** | **PTV/OBV/r + DSV** | **PTV/OBV/r** | **PegIFN + RBV+SOF** |
| --- | --- | --- | --- | --- | --- | --- | --- | --- | --- |
|  |  |  |  |  |  |  |  |  |  |
| **1a** | 12 weeks | 12 weeks | NO | 12 weeks | 12 weeks | 12 weeks | 12 weeks (+RBV) | NO | 12 weeks |
| **1b** | 12 weeks | 12 weeks | NO | 12 weeks | 12 weeks | 12 weeks | 12 weeks | NO | 12 weeks |
| **2** | NO | 12 weeks | 12 weeks | NO | 12 weeks | NO | NO | NO | 12 weeks |
| **3** | NO | 12 weeks | 24 weeks | NO | 12 weeks | NO | NO | NO | 12 weeks |
| **4** | 12 weeks | 12 weeks | NO | 12 weeks | 12 weeks | 12 weeks | NO | 12 weeks (+RBV) | 12 weeks |
| **5,6** | 12 weeks | 12 weeks | NO | NO | 12 weeks | NO | NO | NO | 12 weeks |

b. Chronic HCV with compensated cirrhosis (Child Pugh A)

| **Genotype** | **SOF/LDV** | **SOF + DCV** | **SOF+RBV** | **GZR/EBR** | **SOF/VEL** | **SMV + SOF** | **PTV/OBV/r + DSV** | **PTV/OBV/r** | **PegIFN + RBV+SOF** |
| --- | --- | --- | --- | --- | --- | --- | --- | --- | --- |
|  |  |  |  |  |  |  |  |  |  |
| **1a** | 24 weeks or | 24 weeks or | NO | 12 weeks | 12 weeks | 24 weeks or | 24 weeks (+RBV) | NO | 12 weeks |
|  | 12 weeks( + RBV) | 12 weeks (+RBV) |  |  |  | 12 weeks (+RBV) |  |  |  |
| **1b** | 24 weeks or | 24 weeks or | NO | 12 weeks | 12 weeks | 24 weeks or | 12 weeks (+RBV) | NO | 12 weeks |
|  | 12 weeks( + RBV) | 12 weeks (+RBV) |  |  |  | 12 weeks (+RBV) |  |  |  |
| **2** | NO | 12 weeks | 16 – 20 weeks | NO | 12 weeks | NO | NO | NO | 12 weeks |
| **3** | NO | 24 weeks + RBV | NO | NO | 12 weeks | NO | NO | NO | 12 weeks |
| **4** | 24 weeks or | 24 weeks or | NO | 12 weeks | 12 weeks | 24 weeks or | NO | 24 weeks (+RBV) | 12 weeks |
|  | 12 weeks (+RBV) | 12 weeks (+RBV) |  |  |  | 12 weeks (+RBV) |  |  |  |
| **5,6** | 24 weeks or | 24 weeks or | NO | NO | 12 weeks | NO | NO | NO | 12 weeks |
|  | 12 weeks (+RBV) | 12 weeks (+RBV) |  |  |  |  |  |  |  |

c. Chronic HCV decompensated cirrhosis (including moderate and severe liver failure, Child Pugh B or C).

| Genotype | SOF+RBV | SOF/LDV | SOF/DCV | SOF/VEL |
| --- | --- | --- | --- | --- |
| **1a** | NO | 24 weeks or 12 weeks (+RBV) | 24 weeks or 12 weeks (+ RBV) | 24 weeks or 12 weeks (+ RBV) |
| **1b** | NO |  |  |  |
| **2** | 16-20 weeks | NO |  |  |
| **3** | NO | NO |  |  |
| **4** | NO | 24 weeks or 12 weeks (+RBV) |  |  |
| **5, 6** | NO |  |  |  |

d: Dosage for different Treatment regimens following Decision No. 5012/QĐ-BYT by MoH, Vietnam

| **Name of Drug** | **Characteristic** | **Dosage** | **Available in HTD** |
| --- | --- | --- | --- |
| **Sofosbuvir (SOF)** | Tablet 400mg | One per day | Available |
| **Daclatasvir (DCV)** | Tablet 30mg, 60mg | One per day | Available |
| **Sofosbuvir/ Ledipasvir (LDV)** | Tablet 400mg SOF /90mg LDV | One per day | Available |
| **Sofosbuvir/ Velpatasvir (VEL)** | Tablet 400mg SOF /100mg VEL | One per day | Available |
| **Paritaprevir (PTV)/ Ombitasvir (OBV)/ Ritonavir** | Tablet: 75mg PTV/12,5mgOBV/ 50mg ritonavir | Two per day | Not available |
| **Dasabuvir (DSV)** | Tablet 250mg | Two per day | Not available |
| **Simeprevir (SMV)** | Capsule 150 mg | One per day | Not available |
| **Grazoprevir(GZR)/Elbasvir (EBR)** | Tablet 100mg GZR /elbasvir 50mg EBR | One per day | Available |

SOF; Sofosbuvir, DCV; Daclatasvir, LDV; Ledipasvir, VEL; Velpatasvir, PTV; Paritaprevir, OBV ; Ombitasvir, r ; Ritonavir, DSV; Dasabuvir, SMV; Simeprevir, GZR; Grazoprevir, EBR; Elbasvir, PegIFN; Pegylated interferon, RBV; Ribavirin, NO; Not recommended.
