# Supplementary material for: Effectiveness of sofosbuvir based direct-acting antiviral regimens for chronic hepatitis C virus genotype 6 patients: Real-world experience in Vietnam
Source: PLoS One. 2020 May 20;15(5):e0233446. doi: 10.1371/journal.pone.0233446 (PMC7239434; doi:10.1371/journal.pone.0233446)
Supplement: S2 Table — (DOCX) [file pone.0233446.s002.docx]

S2 File. Test recommendation before, during and after treatment of Chronic HCV with DAA, DAA+RBV and PegINF+RBV+SOF (Issued together with Decision No. 5012/QĐ-BYT by MoH, Vietnam)

| **Time** | **DAA** | | | | | **DAA+RBV** | | | | | **PegIFN + RBV + SOF** | | | | | |
| --- | --- | --- | --- | --- | --- | --- | --- | --- | --- | --- | --- | --- | --- | --- | --- | --- |
|  | FBC, renal, liver function | Abdominal ultrasound, AFP | Evaluation of liver fibrosis ^$^ | HCV RNA | HIV, HBsAg, HCV genotype | FBC, renal, liver function | Abdominal ultrasound, AFP | Evaluation of liver fibrosis ^$^ | HCV RNA* | HIV, HBsAg, HCV genotype | FBC, renal, liver function | Thyroid function | Abdominal ultrasound, Chest X ray , ECG, AFP | Evaluation of liver fibrosis ^$^ | HCV RNA | HBsAg, HIV, HCV genotype |
| Baseline | X | X | X | X | X | X | X | X | X | X | X | X | X | X | X | X |
| Week 4 | X |  |  | X |  | X |  |  | X |  | X | X |  |  | X |  |
| Week 8 |  |  |  | X* |  | X |  |  | X* |  | X |  |  |  | X* |  |
| Week 12 | X | X |  |  |  | X | X |  |  |  | X | X | X |  |  |  |
| 12 Week after EOT | X | X | X | X |  | X | X | X | X |  | X | X | X |  | X |  |
| 24 Week after EOT |  | X |  | X |  |  | X |  | X |  |  | X |  | X | X |  |

FBC (Full blood count); (Red blood cell count, white blood cell counts, Platelet count, HCT, MCV, MCH, MCHC,RDW hemoglobin), Renal function test; (blood urea, creatinine) Liver function test (Bilirubin, AST, ALT, GGT, AFP, albumin, glucose, prothrombin), Thyroid function test; (T3, T4 ?)

* If HCV RNA at week 4 of treatment is above the detection threshold, repeat HCV RNA test at week 8. If HCV RNA increases more than 1log10 IU/ml: treatment should be stopped. FBC; Full Blood Count, ^$^; Noninvasive method, AFP; Alpha-fetoprotein, ECG; Electrocardiogram, EOT; End of Treatment
